# Chemical Composition of a Supercritical Fluid (Sfe-CO_2_) Extract from *Baeckea frutescens* L. Leaves and Its Bioactivity Against Two Pathogenic Fungi Isolated from the Tea Plant (*Camellia sinensis* (L.) O. Kuntze)

**DOI:** 10.3390/plants9091119

**Published:** 2020-08-29

**Authors:** Hao Jiang, Mengting Zhang, Li Qin, Dongxu Wang, Feng Yu, Wenhui Liang, Chuankui Song, Daniel Granato

**Affiliations:** 1 State Key Laboratory of Tea Plant Biology and Utilization, Anhui Agricultural University, 130 West Changjiang Road, Hefei 230036, China or ahjh88@163.com (H.J.); zhangmt@ahau.edu.cn (M.Z.); andyfdyx@ahau.edu.cn (F.Y.); 2 Anhui Provincial Key Laboratory of Microbial Pest Control, Anhui Agricultural University, 130 West Changjiang Road, Hefei 230036, China; qinli@ahau.edu.cn; 3 School of Grain Science and Technology, Jiangsu University of Science and Technology, Zhenjiang 212003, China; wdx@just.edu.cn; 4 Guangxi Forestry Research Institute, Guangxi Engineering and Technology Research Center for Woody Spices, Guangxi Key Laboratory for Cultivation and Utilization of Special Non-Timber Forest Crops, 23 Yongwu Road, Nanning 530002, China; L.wenhui@163.com; 5 Food Processing and Quality, Natural Resources Institute Finland, Tietotie 2, 02150 Espoo, Finland

**Keywords:** *Camellia sinensis*, pathogenic fungi, *Pseudopestalotiopsis camelliae-sinensis*, Colletotrichum gloeosporioides, supercritical fluid extraction, antifungal activity, tea tree plant diseases, *Baeckea frutescens*

## Abstract

*Colletotrichum gloeosporioides* and *Pseudopestalotiopsis camelliae-sinensis* are the two most important tea plant (*Camellia sinensis* L.) pathogenic fungi. Interest in natural plant extracts as alternatives to synthetic chemical fungicides to control plant pathogens is growing. In this study, the volatile fraction of *Baeckea frutescens* L. was extracted by supercritical fluid extraction (SFE-CO_2_), and its chemical composition was analyzed, and investigated for its antifungal activity against *C. gloeosporioides* and *P. camelliae*. The major constituents of the volatile fraction were β-caryophyllene (28.05%), α-caryophyllene (24.02%), δ-cadinene (6.29%) and eucalyptol (5.46%) in *B. frutescens* SFE-CO_2_ extracts. The terpineol, linalool, terpinen-4-ol and eucalyptol showed strong contact antifungal activity against *P. camelliae* and *C. gloeosporioides* with median inhibitory concentration (MIC_50_) in the range of 0.69 μL/mL to 2.79 μL/mL and 0.62 μL/mL to 2.18 μL/mL, respectively. Additionally, the volatile fraction had high fumigation antifungal activity against *P. camelliae* and *C. gloeosporioides* with an inhibition rate between 20.87% and 92.91%. Terpineol presented the highest antifungal activity in the contact and fumigation toxicity assays. Terpineol, linalool, terpinen-4-ol and eucalyptol were associated with the most active chemical compounds in the volatile fraction against the fungi. The results suggest that *B. frutescens* SFE-CO_2_ extracts are potential ingredients to develop a natural fungicide for control of tea plant pathogens.

## 1. Introduction

As an alternative to using chemical fungicides, plant extracts have attracted the attention of chemical companies as they may be used as botanical fungicides. *Baeckea frutescens* L. is an important medicinal plant belonging to the Myrtaceae family [1] and is found in Peninsular Malaysia and Sumatra and distributed along coastal areas of southeastern China and Australia [2]. *B. frutescens* has various bioactive properties, such as antibacterial [1,3], antioxidant [2], anticancer [4], anti-inflammatory [5] and insecticidal activities [1]. The leaves of *B. frutescens* are rich in various volatile constituents such as terpenoids, sesquiterpenes and phenylpropanoids, which are secondary metabolites associated with numerous bioactivities [1,5,6].

The tea plant (*Camellia sinensis* L.) is a crucial commercial crop in world [7]. Tea, made from fresh leaves of *C. sinensis*, is a prominent beverage worldwide and is the second most consumed nonalcoholic beverage after water [8]. The tea plant suffers from biotic stresses of some pathogenic fungi, which is a serious concern for the tea industry as this condition impacts on reduced tea yields, decreased quality and damaged human health [9,10]. Several *Pestalotiopsis*-like species are common phytopathogens that cause tea grey blight disease and result in severe losses (10 to 20%) in tea production [11,12]. *Pseudopestalotiopsis camelliae-sinensis* was shown to cause grey blight disease on tea plants in China [13]. Some research has showed that planting disease-resistant varieties of *C. sinensis* has a certain effect on controlling the tea plant disease infected by *Pestalotiopsis* species [14]. In addition, the *Colletotrichum* species is also a common group of plant pathogens that are responsible for anthracnose diseases [15,16]. *Colletotrichum gloeosporioides* could lead to tea anthracnose disease, which a is serious foliar disease of the tea plant and causes severe damage accompanied by high yield losses [17]. Meanwhile, *C. gloeosporioides*-contaminated tea beverage may be harmful to human health [10]. It was recently reported that the leaves of tea plants infected by *C. gloeosporioides* were reduced in 30–60% of the tea products [18,19]. Altogether, *P. camelliae and C. gloeosporioides* are two of most destructive pathogens of foliar disease on tea plants and strategies to inhibit their growth are highly necessary.

At present, synthetic chemical fungicides are still the primary control approach to prevent fungal-related diseases in tea leaves. However, the indiscriminate use of these chemical fungicides not only reduces the quality of tea products and causes tea security crises, but also represents a harmful condition for human health and the natural environment [20,21,22]. Nowadays, effective and environmentally safe plant protection products which protect against phytopathogenic fungi are urgently needed in modern agriculture [23,24]. Therefore, replacing synthetic chemical fungicides by botanical alternatives could be a suitable choice to avoid toxicological and environmental-related issues. For instance, the botanical secondary metabolites, such as alkaloids [25], flavonoids [26] and volatile organic compounds [27], may act as antibacterial active substances to inhibit plant diseases. Therefore, botanical fungicides have been considered as environmentally friendly alternatives to synthetic chemical fungicides.

**Figure 1:**
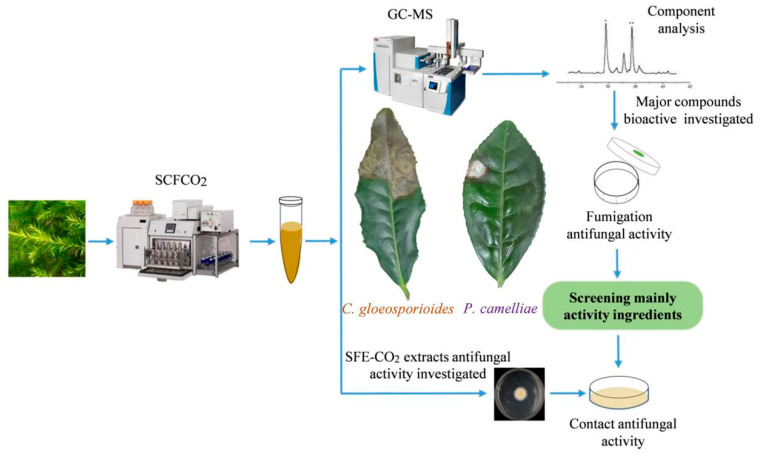
The mainly schematic diagram of the research process.

Traditionally, the volatile fraction of plants is extracted by steam distillation and organic solvent extraction. Supercritical fluid extraction (SFE) utilizes supercritical fluids, which exhibit liquid-like as well as gas-like properties, and has become an effective method for separating and extracting more apolar compounds, as well as the extraction of polyphenols, lipids, and essential oils [28,29,30]. Furthermore, conventional extraction with organic solvents has some disadvantages, such as high energy costs, and the possible loss of volatile compounds during the removal of the solvent [31]. Therefore, supercritical fluids are attractive for extracting flavors present in natural materials [28]. The supercritical fluid extraction with carbon dioxide (SFE-CO_2_), a green extraction method, provides the final product without organic solvent residues [32]. Due to the properties of CO_2_, such as being odorless, colorless, safe, nontoxic and recyclable [28], SFE-CO_2_ has been widely used for the extraction of volatile compounds [28]. To the best of our knowledge, there are still no reports on the chemical composition of the supercritical fluid (SFE-CO_2_) extract of *B. frutescens* leaves and its bioactivity against *P. camelliae* and *C. gloeosporioides*. Therefore, the aims of this study were to investigate the chemical composition of the SFE-CO_2_ extract from *B. frutescens* using GC-MS. In addition, the antifungal effects of SFE-CO_2_ extract from *B. frutescens* against *P. camelliae* and *C. gloeosporioides* were also evaluated. The main research outline applied in this study is shown in Figure 1.

## 2. Results and Discussion

### 2.1. Chemical Composition of the B. Frutescens Leaf SFE-CO2 Extract

The yield of the extract obtained by SFE-CO_2_ was 2.2% (*w/w* relative to dry material weight). The major compounds of the SFE-CO_2_ extract of were analyzed using GC-MS, and the peaks with matching similarly of more than 80% were accepted as candidate compounds. These compounds were further confirmed and identified by comparing the mass spectra with those from National Institute of Standards and Technology (NIST)mass spectral (http://webbook.nist.gov/chemistry/) and National Institutes of Health (NIH) databases, as well as by calculating the retention index (RI) and comparing RI from some of the previous literature records. Some major compounds were identified by co-injection of available standard compounds, such as α-pinene, β-pinene, eucalyptol, γ-terpinene, linalool, terpinen-4-ol, terpineol, β-caryophyllene and α-caryophyllene. The GC-MS separation of SFE-CO_2_ extract is shown in supplementary material Figure S1.The chemical composition of the SFE-CO_2_ extract is listed in Table 1, along with their retention indices, relative contents and identification.

As observed in Table 1, a total of 27 compounds were identified in the SFE-CO_2_ extract, accounting for 98.99% of total volatile oil. The major constituents of the volatile SFE-CO_2_ extract were β-caryophyllene (28.05%), α-caryophyllene (24.02%), δ-cadinene (6.29%), eucalyptol (5.46%) and β-pinene (5.21%), followed by terpineol (4.54%), cubenol (2.81%) and γ-terpinene (2.49%). The volatile organic metabolites from *B. frutescens* have been investigated elsewhere and α-pinene, β-caryophyllene, γ-terpinene, terpineol, 1,8-cineole and linalool were the main components of *B. frutescens* volatile oils [1,33]. Furtnemore, Myrtaceae was divided into different chemotypes, according to the single most abundant compound, called dominant terpenes, in its plant volatile oils [33,34]. In most cases, 1,8-cineole or α-pinene are the most abundant and most common terpenes compounds in the majority of Myrtaceae foliar terpene profile, so in the vast majority of species (80%) there is an α-pinene or 1,8-cineole foliar terpene chemotype [34]. In this study, β-caryophyllene and α-caryophyllene were the most abundant terpene compounds. Compared with previous observations, it may represent a new or another chemical type of *B. frutescens* based on the main chemical composition of the volatile oil.

The hydrodistillation volatile oil of *B. frutescens* leaves grown in Vietnam was found to contain β-pinene (19.0%), γ-terpinene (11.7%), α-pinene (11.1%) and (E)-caryophyllene (7.1%) [1]. Similarly, the hydrodistillation volatile oil of *B. frutescens* leaves collected in Vietnam was shown to contain α-humulene (19.2%), β-caryophyllene (17.3%), baeckeol (13.8%), α-thujene (8.8%), linalool (5.6%) and eucalyptol (5.6%) [33]. From these results, it seems the essential oil composition of *B. frutescens* leaves exhibits high chemical variability [35]. Several factors may be related to these differences, such as geographic location, harvest time, local climate and the plant physiological status. In addition, the method of essential oil extraction also becomes an important factor. The *B. frutescens* volatile essential oil obtained by solid-phase micro extraction (SPME) presented γ-terpinene, o-cymene, α-pinene and Eucalyptol as major compounds. However, head-space extraction (HS) and conventional hydro distillation (HD) detected β-pinene, γ-terpinene, α-pinene and o-cymene as major compounds [33]. Meanwhile, the particle size of plant material is also an important factor in SFE and HD [28,36]. Some results reported that decrease of particle size increased the extract yield and extraction rate [28,36]. The above findings suggest that further studies on plant materials and extraction methods are needed.

This is the first report in which the chemical composition of a supercritical fluid (SFE-CO_2_) extract of *B. frutescens* leaves is provided. β-Caryophyllene, α-caryophyllene, δ-cadinene and eucalyptol were the major components.

### 2.2. Contact Antifungal Activity of B. frutescens SFE-CO_2_ Extract

**Figure 2:**
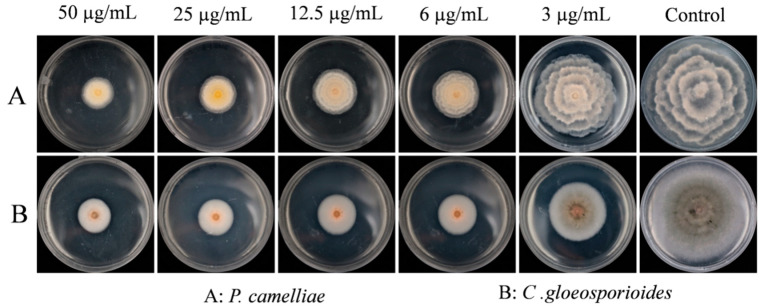
The mycelial growth of *P. camelliae* (**A**) and *C. gloeosporioides* (**B**) on different concentration of toxic potato dextrose agar medium (PDA) medium after 7 days.

The SFE-CO_2_ extract of *B. frutescens* leaves exhibited an inhibitory effect on mycelial growth of two tea plant pathogenic fungi as shown in Figure 2. The mycelial growth of *P. camelliae* and *C. gloeosporioides* was strongly inhibited and this effect was dose-dependent as shown in Figure 2 and Table 2.

As seen in Figure 2, from concentrations of 6 μg/mL to 50 μg/mL, the SFE-CO_2_ extract of *B. frutescens* leaves showed a significantly limited mycelial growth of both fungi. In addition, the extract presented stronger inhibited activity against *C. gloeosporioides* than against *P. camelliae* (Figure 2) as also observed by the lower MIC_50_ value for *C. gloeosporioides*.

The median inhibitory concentration (MIC_50_) of the *B. frutescens* leaf SFE-CO_2_ extract against two phytopathogenics was also evaluated (Table 2). The inhibitory activity of *B. frutescens* SFE-CO_2_ extract against *C. gloeosporioides* exhibited the lowest MIC_50_ value of 4.79 µg/mL, while the MIC_50_ against *P. camelliae* was 5.11 µg/mL. However, compared with other previously published papers, our research exhibited that the *B. frutescens* SFE-CO2 extracts have stronger antifungal activity. For example, three plants (oregano, thyme and ajwain) SFE-CO_2_ extracts showed broad-spectrum antifungal activity against four *Aspergillus* species fungi with MIC values in the range of 128–1024 µg/mL [37]. In addition, the SFE-CO_2_ extract of *Prunus persica* leaves presented MIC_50_ of 62.50 µg/mL against *Candida albicans* [38]. In our study, the *B. frutescens* SFE-CO_2_ extract revealed a stronger antifungal activity against two fungi (MIC_50_ values of 4.79 µg/mL and 5.11 µg/mL, respectively). Overall, results showed that *C. gloeosporioides* may be the most sensitive micro-organism for *B. frutescens* SFE-CO_2_ extract.

### 2.3. Screening for Fumigation Antifungal Activities of Major Chemical Compounds

**Figure 3:**
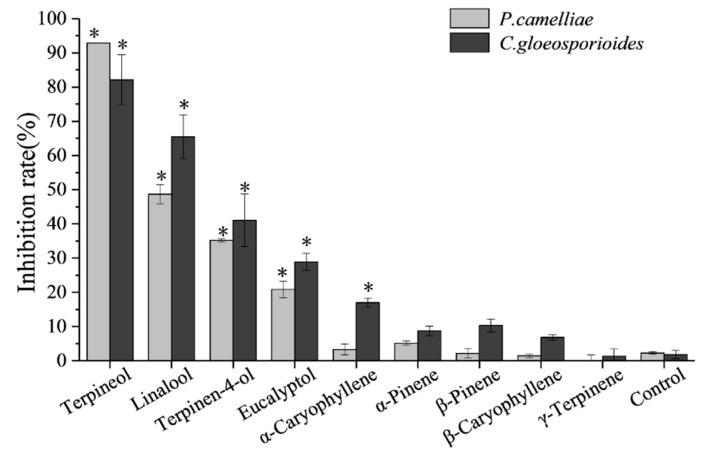
Fumigation activity of the ten main constituents available against *P. camelliae* and *C. gloeosporioides* at 200 μL/L air after 5 days. * Data are presented as mean (±standard error) representative of significantly different versus control group at *p* ≤ 0.05 (Duncan’s test).

In order to quickly screen the antifungal activity of the nine major chemical compounds present in the SFE-CO_2_ extract the fumigation method against the mycelial growth of *P. camelliae* and *C. gloeosporioides* was used. The results are presented in Figure 3.

**Figure 4:**
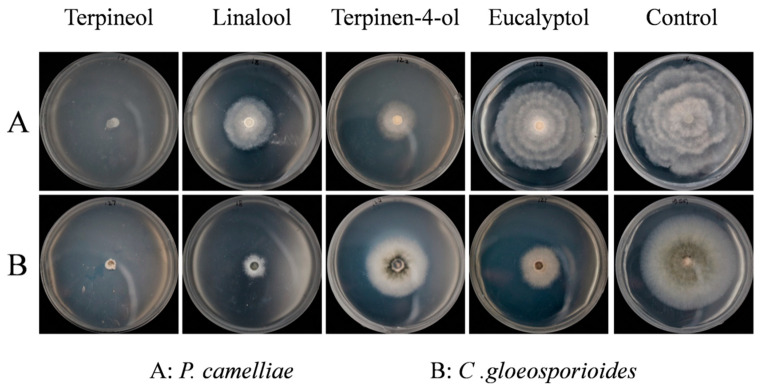
Fumigation activity of five mainly constituents on mycelial growth of *P. camelliae* (**A**) and *C. gloeosporioides* (**B**) at 200 μL/L air of concentration after 5 days, respectively.

In Figure 3, it can be seen that the four primary compounds (terpineol, linalool, terpinen-4-ol and eucalyptol) had stronger fumigation activity against *P. camelliae* and *C. gloeosporioides*, respectively. By contrast, the other five major compounds (α-pinene, β-pinene, γ-terpinene, β-caryophyllene and α-caryophyllene) only exhibited weak fumigation activity against two tea plant pathogenic fungi, respectively. In the fumigation activity assay, the mycelial growth of *P. camelliae* and *C. gloeosporioides* was inhibited by the four relatively higher activity standards (terpineol, linalool, terpinen-4-ol and eucalyptol) as shown in Figure 4.

As shown in Figure 3 and Figure 4, terpineol showed the strongest fumigation inhibitory effects against *P. camelliae*, presenting an inhibition rate of 92.91%, followed by linalool, terpinen-4-ol and eucalyptol which inhibited 48.68%, 35.20% and 20.87%, respectively. Terpineol also exhibited the highest fumigation inhibitory effect (82.15%) against *C. gloeosporioides*, followed by linalool, terpinen-4-ol and eucalyptol that inhibited 65.51%, 41.06% and 28.92%, respectively. In addition, α-caryophyllene showed weak fumigation activity (16.93%) against *C. gloeosporioides*. In a previous report, linalool showed fumigation inhibitory effect (83.7% inhibition) against, *Aspergillus ochraceus* at an air concentration of 56 µg/mL [39].

However, as seen in Figure 3, the other five major compounds (α-pinene, β-pinene, γ-terpinene, β-caryophyllene and α-caryophyllene) showed less than 17% inhibition rate against mycelial growth of *P. camelliae* and *C. gloeosporioides*, respectively. These results are in-line with a previous report that showed that α-pinene, β-pinene and β-caryophyllene did not present fumigation inhibitory effects against *Aspergillus ochraceus*, *A. flavus*, and *A. niger* at an air concentration of 56 µg/mL [39].

Conversely, many plant essential oils have been evaluated for fumigation activity against plant pathogens [39,40]. For example, the antifungal activity of cinnamon oil, clove oil, eugenol and geraniol was dose-dependent, and relatively higher doses (8 μL/disc) presented stronger antifungal activities [41]. At a concentration of 10 μL/L air, cinnamon oil, cinnamon bark oil, *Litsea cubeba* oil, *Angelica dahurica* oil and thyme oil showed high fumigation inhibitory effects (inhibition rate of 100%) against *Villosiclava virens* [40]. *Trans*-cinnamaldehyde, the major component of cinnamon oil and cinnamon bark oil, exhibited strong fumigation activity against *Villosiclava virens* with an effective medium concentration EC_50_ of 0.5 µL/L air [40].

### 2.4. Contact Antifungal Activity of Major Activity Components

**Figure 5:**
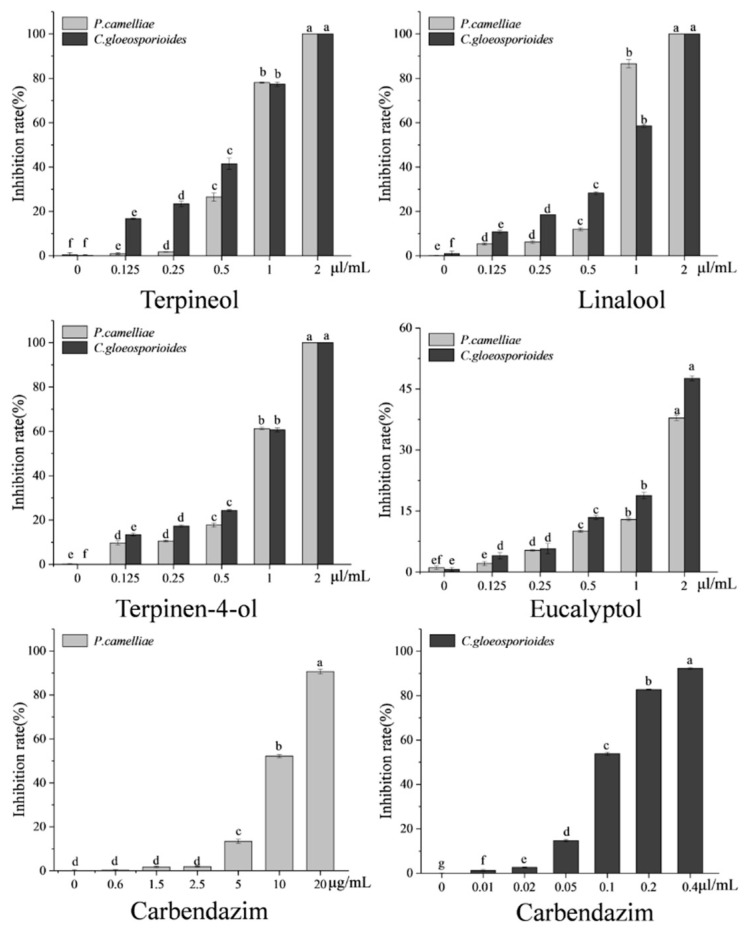
The contact antifungal activity of four major compounds and carbendazim (positive control) against *P. camelliae* and *C. gloeosporioides* after 7 days. Percentage values followed by the same letter are not significantly different in the same group at *p* ≤ 0.05 (Duncan’s test).

According to the results of the fumigation activity assay, the four compounds with the highest activity (terpineol, linalool, terpinen-4-ol and eucalyptol) were thoroughly evaluated in relation to their contact antifungal activity against *P. camelliae* and *C. gloeosporioides*. The results of contact antifungal activity of the chemical compounds compared to that of carbendazin are presented in Figure 5. The contact antifungal toxicity of terpineol, linalool, terpinen-4-ol and eucalyptol against *P. camelliae* and *C. gloeosporioides* was concentration-dependent.

**Figure 6:**
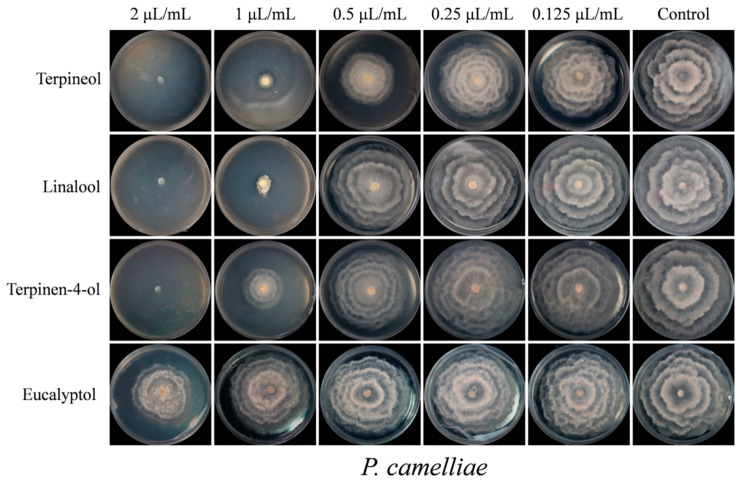
The mycelial growth of *P. camelliae* treatment by four mainly activity compounds with contact antifungal activity after 7 days.

Terpineol, linalool, terpinen-4-ol and eucalyptol showed inhibitory effects on mycelial growth of *P. camelliae* at the five different concentrations, Figure 6. As shown in Figure 5 and Figure 6, at 2 µL/mL, terpineol, linalool and terpinen-4-ol exhibited the strongest contact activity with 100% inhibition of *P. camelliae*. In contrast, at 20 µL/mL, the commercial fungicide carbendazim inhibited 90.64% the growth of *P. camelliae* (Figure 5). Linalool, terpineol and terpinen-4-ol exhibited the highest inhibitory effects on the mycelial growth of *P. camelliae* at 1 µL/mL (Figure 6). Terpineol presented antifungal activity (26.48% inhibition) at 0.5 µL/mL (Figure 6), whereas eucalyptol showed moderate antifungal activity (37.9% inhibition) against *P. camelliae* at 2 µL/mL. In addition, compared with the control, other concentrations of the linalool and terpinen-4-ol also showed significantly contact antifungal activity (Figure 5). Eucalyptol also showed antifungal activity and inhibited mycelial growth of *P. camelliae* at concentrations higher than 0.25 µL/mL.

**Figure 7:**
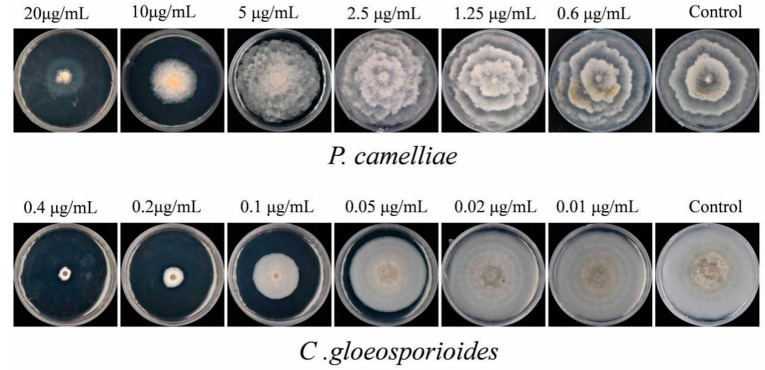
The mycelial growth of *C. gloeosporioides* and *P. camelliae* treatment by commercial fungicide carbendazim (positive control) with contact antifungal activity after 7 days.

However, as expected, carbendazim showed a strong inhibitory effect on the mycelial growth of *P. camelliae* at concentrations higher than 10 µL/mL (Figure 7). Carbendazim presented the highest antifungal activity (90.64% inhibition) at 20 µL/mL, and it also showed moderate antifungal activity with inhibition rate of 52.17% at 10 µL/mL (Figure 5 and Figure 7). In summary, compared with carbendazim, the tested compounds (terpineol, linalool, terpinen-4-ol and eucalyptol) showed stronger antifungal activity and inhibited mycelial growth of *P. camelliae*.

**Figure 8:**
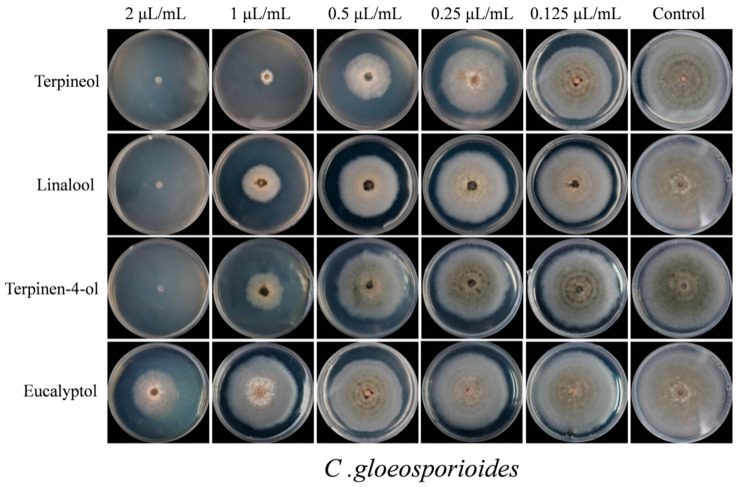
The mycelial growth of *C. gloeosporioides* after treatment by four mainly activity compounds with contact antifungal activity after 7 days.

The effects of terpineol, linalool, terpinen-4-ol and eucalyptol on the mycelial growth of *C. gloeosporioides* at the five different concentrations are shown in Figure 8. Compared with the control, all the four chemical compounds had significant difference in the contact antifungal activity and inhibition of mycelial growth of *C. gloeosporioides* (Figure 5 and Figure 8). The results show that terpineol, linalool and terpinen-4-ol were able to inhibit 100% of mycelial growth (Figure 8) and at 2 µL/mL it inhibited 100% of *C. gloeosporioides* growth. On the other hand, eucalyptol inhibited 47.63% of the *C. gloeosporioides* growth (Figure 5). Terpineol showed the strongest contact inhibition of *C. gloeosporioides* mycelial growth, followed by linalool, terpinen-4-ol and eucalyptol (Figure 8). However, compared with the tested four compounds, carbendazim exhibited the strongest contact antifungal activity and inhibition of mycelial growth of *C. gloeosporioides* (Figure 5 and Figure 7). Carbendazim exhibited the strongest contact activity with 92.27% inhibition of *C. gloeosporioides* at 0.4 µL/mL (Figure 5). In addition, at 0.2 µL/mL and 0.1 µL/mL, carbendazim also inhibited 82.76% and 53.8%of *C. gloeosporioides* mycelial growth, respectively (Figure 7).

This result is in-line with other studies that showed that the treatment with an 8 μL/disc of linalool dramatically decreased the conidial germination of *C. gloeosporioides* to 3.4%, but eugenol showed moderate anti-germination activities with the conidial germination of *C. gloeosporioides* was 50.4% [41]. At 1 µL/mL, terpineol, linalool, and terpinen-4-ol inhibitedthe mycelial growth by 77.42%, 58.53% and 60.62%, respectively. However, eucalyptol at 1 µL/mL showed relatively weak activity (<20% inhibition) in relation to the mycelial growth of *C. gloeosporioides* [41].

The median inhibitory concentration (MIC_50_) values of terpineol, linalool, terpinen-4-ol and eucalyptol compounds were estimated using the logistic analyses model and the results are displayed in Table 3.

The high determination coefficient values (R^2^ ≥ 0.90) indicate that the logistic analyses model is suitable to fit the antimicrobial data. Terpineol, linalool, terpinen-4-ol and eucalyptol showed strong contact antifungal activity against *P. camelliae* with remarkable MIC_50_ values of 0.69, 0.73, 0.86 and 2.79μL/mL, respectively (Table 3). Compared with the commercial fungicide carbendazim (MIC_50_ values of 9.70 μL/mL), terpineol, linalool, terpinen-4-ol and eucalyptol had 3.5 to 14-fold more antifungal activity against *P. camelliae* (Table 3). Terpineol, linalool, terpinen-4-ol and eucalyptol also exhibited strong inhibitory effect against *C. gloeosporioides*, with MIC_50_ values of 0.62, 0.93, 0.94 and 2.18 μL/mL, respectively (Table 3). The carbendazim showed acute contact antifungal activity (MIC_50_ = 0.095 μL/mL) in relation to *C. gloeosporioides*. Overall, terpineol had the highest antifungal activity against *P. camelliae* and *C. gloeosporioides*, followed by linalool, terpinen-4-ol and eucalyptol. This observation is consistent with other research stating that linalool and terpinen-4-ol were more active than eucalyptol [42,43]. In fact, the contact antifungal activity of terpineol was approximately four times higher than that of eucalyptol against *P. camelliae* and *C. gloeosporioides*.

Some terpenoids, such as terpineol, linalool, terpinen-4-ol and eucalyptol have antifungal activity against plant pathogens. For example, terpineol could strongly inhibit the mycelial growth of *Penicillium digitatum*, with the minimum inhibitory concentration of 2.00 µL/mL [44]. Terpinen-4-ol and eucalyptol exhibited antifungal activity against *Candida albicans* with MIC_50_ values of 0.06% (*v*/*v*) and 4% (*v*/*v*), respectively [42]. Linalool and eucalyptol inhibited the mycelial growth of *Fusarium oxysporum*, *Fusarium solani*, and *Cylindrocarpon destrutans* [45], whereas capsidiol exhibited antifungal activity against *C. gloeosporioides* [46]. Regarding plant essential oils, the antifungal activity against *C. gloeosporioides* or *P. camelliae* has also been reported. For instance, *Engenia caryophyllus* and *Cinnamomum cassia* essential oils showed good contact antifungal toxicity against *C. gloeosporioides*, with 100 and 189 μL/L, respectively [47]. Cinnamon oil (8 μL/disc) exhibited strong contact antifungal efficacies against *C. gloeosporioides* [41]. Thus, it seems that our samples are very effective against plant pathogens compared with other plants.

In general, the fumigation and contact antifungal activity of plant extracts could not be easily correlated with one specific component. In this study, terpineol, linalool, terpinen-4-ol and eucalyptol showed strong fumigation antifungal activity against *P. camelliae* and *C. gloeosporioides* with inhibition rates in the range of 20.87% and 92.91%. Additionally, terpineol, linalool, terpinen-4-ol and eucalyptol also exhibited notable contact antifungal activity against *P. camelliae* and *C. gloeosporioides* with MIC_50_ values in the range of 0.62 μL/mL to 2.79 μL/mL. The commercial fungicide carbendazim showed significantly contact antifungal activity against *P. camelliae* and *C. gloeosporioides* with MIC_50_ values of 0.095 μL/mL and 9.70 μL/mL, respectively. Therefore, it seems that terpineol, linalool, terpinen-4-ol and eucalyptol could be considered as the main active ingredients and are responsible for antifungal activities of the SFE-CO_2_ extract from *B. frutescens* leaves.

## 3. Materials and Methods

### 3.1. Plant Materials

The leaves of *Baeckea frutescens* were collected at the Guangxi Forestry Research Institute, Nanning, Guangxi Province, China in September of 2018. The *B. frutescens* trees were cultivated in the resource garden of Guangxi Forestry Research Institute. *B. frutescens* were authenticated by Prof. Wen-Hui Liang (Guangxi Forestry Research Institute). A voucher specimen (No. GX201816) has been deposited in the State Key Laboratory of Tea Plant Biology and Utilization, Anhui Agricultural University. A total of 2160 g *B. frutescens* leaves were collected from nine randomly selected trees. The leaves were freeze-dried in a freeze dryer (model Martin Christ ALPHA 1-4 LD, Osterode am Harz, Germany). The dried leaves (10 mm × 1 mm, 4% humidity) were ground into powder (filtered through a 200-mesh sieve) and stored at −20 °C.

### 3.2. Chemicals

The standard chemicals—α-pinene, β-pinene, eucalyptol (1.8-cineol), γ-terpinene, linalool, terpinen-4-ol, terpineol, β-caryophyllene and α-caryophyllene—with a purity of ≥99% were purchased from Sigma Chemical Co. (Fairfield, OH, USA). The homologous series of (C5–C36) alkanes were purchased from Sigma Chemical Co. (Fairfield, OH, USA). HPLC-grade methanol and N-hexane were obtained from Fisher Scientific (Fair Lawn, NJ, USA). Ultrapure water was used in the experiments (Purelab Plus, Pall, Show Low, AZ, USA). All other chemicals were of analytical grade and purchased from Beijing Chemical Works (Beijing, China).

### 3.3. Isolation and Identification of Fungal Pathogens

Diseased tea leaves were collected from the tea plantation located in Cuona County, Shannan city, Tibet Autonomous Region in China. The pathogenic fungi were isolated from diseased leaves that presented visible grey blight disease and anthracnose symptoms using traditional method as previously described [12,48] and with slight modification. Briefly, the symptomatic leaves were surface-sterilized in 1% NaClO for 2 min, then in 70% ethanol for 1 min, rinsed three times in sterile water, and then margins of lesions were cut into small pieces and transferred into potato dextrose agar medium (PDA, Difco Company). The culture was incubated at 25 °C for 3 to 5 days until fungal hyphae started to grow from the pieces. Single-hyphal tip was transferred to a new PDA plate to purify isolates and two strains were obtained. To identify the two fungal isolates, DNA was extracted, amplified, and sequenced using universal primers (Internal Transcribed Spacer, ITS1/ITS4). BLAST (Basic Local Alignment Search Tool) analysis of GenBank data showed 100% sequence homology with the ITS sequence of strain for *Colletotrichum gloeosporioides* (Accession No. KF836743.1) and *Pseudopestalotiopsis camelliae-sinensis* (Accession No. MK909901.1), respectively. The two pathogenic fungi *C. gloeosporioides* and *P. camelliae* cultured using potato dextrose agar medium at 4 °C and stored for future use.

### 3.4. Supercritical Fluid (SFE-CO2) Extract of Baeckea frutescens *L.*

The extraction was performed with a laboratory scale multi-vessel accelerated supercritical fluid extraction system (model MV-10 ASFE Waters Corporation, Milford, MA, USA). The dried *B. frutescens* leaf powder (5.0 g) was placed in a 25 mL supercritical fluid extractor vessel and was extracted using supercritical carbon dioxide without any cosolvents. The temperature and pressure were set at 40 °C and 180 bar, respectively. The extract laden Supercritical CO_2_ (flow rate 13 min/mL) was sent to extractor vessel through a pressure pump for 70 min, with 20 min dynamic duration, 30 min of static duration and 20 min of dynamic duration, respectively. The extraction efficiency (yield) was 2.2% (110 mg), and the SFE-CO_2_ extract was stored for future use.

### 3.5. Contact Antifungal Activities Bioassay

The contact antifungal activity of the SFE-CO_2_ extract was determined using the toxic medium method. Median inhibitory concentration (MIC_50_) of the extract was determined using a serial two-fold micro dilution method against the tea plant pathogenic fungi. The stock solution (100 mg/mL) was serially diluted in 30 mL PDA medium at 45–50 °C and mixed to provide different concentrations (50 µg/mL, 25 µg/mL, 12.5 µg/mL, 6 µg/mL, 3 µg/mL) to evaluate inhibitory activities against *C. gloeosporioides* and *P. camelliae*. Based on preliminary screening activities, the four individual chemical components with higher antifungal activity were determined, and which were adjusted to a series of concentration gradients medium (2.0 μL/mL, 1.0 μL/mL, 0.5 μL/mL, 0.25 μL/mL and 0.125 μL/mL) to evaluate inhibitory activities against *C. gloeosporioides* and *P. camelliae*. Carbendazim, a broad-spectrum fungicide, was used as a positive control and different concentrations were tested. The negative control received the same quantity of acetone mixed with PDA. The 10 mL toxic medium were poured onto aseptic 9 cm plastic Petri dishes. A 5 mm diameter fungal disc of *C. gloeosporioides* or *P. camelliae* was immediately inoculated in the center of each PDA plate and plates were incubated in the dark at 25 °C.

After 7 days of incubation at 25 °C in the dark, the colony growth diameter (mm) was measured using a digital caliper. Each test was repeated three times. The growth inhibition was calculated using the formula.

Inhibition rate (%) = (Dc − Dt)/Dc × 100,(1)

where: D_C_, D_T_—average diameter (mm) of the fungal colony of the control and the treatment, respectively.

### 3.6. Fumigation Antifungal Activities Bioassay

The fumigation activity of the SFE-CO_2_ extract was determined as previously described [49] and with slight modifications. A 5 mm diameter disc of *C. gloeosporioides* or *P. camelliae* was inoculated in the center of each PDA plate (9 cm, the volume about 60 mL air spaces), and a 6 mm filter paper containing 10 μL of the extract or isolated substance was placed on the center of the inner surface of the Petri dish lid. Plastic Petri dishes (60 mL air spaces) offer 50 mL air spaces after the addition of 10 mL PDA medium, and the final concentration was 200 μL/L air. The negative control was composed of acetone. The plate was immediately sealed with parafilm to prevent any leakage of the standards. Three replicates were used for each concentration, and the PDA plates were placed upside down in the incubator. After 5 days of incubation at 25 °C in the dark, the colony growth diameter (mm) was measured using a digital caliper. The growth inhibition was calculated using the above Formula (1).

### 3.7. B. frutescens Extract GC-MS Analysis

The constituents SFE-CO_2_ Extract of *B. frutescens* were analyzed by Thermo Fisher trace 1300 gas chromatography system, equipped with ISQ 7000 MS (Thermo Fisher Scientific, San Jose, CA, USA). Briefly, the stock solution of SFE-CO_2_ extract (10 uL) or the isolated compounds (terpineol, linalool, terpinen-4-ol, eucalyptol, α-caryophllene, (1R)-(+)- α-pinene, (1S)-(-)- α-pinene, α-phellandrene, β-caryophyllene, γ-terpinene) were diluted with n-hexane (1 mL). A DB-5MS column (60 m × 0.25 mm, film thickness 0.25 μm, J&W Scientific, Folsom, CA, USA) was used in the separation of chemical compounds. Helium ( >99.99%) was used as the carrier gas at a flow rate of 1 mL/min. The injector temperature was 250 °C using a splitless injection mode with a sampling time of 1.00 min. The oven program was set as follows: Temperatures and times of 50 °C (5 min), 20 °C/min to 180 °C (5 min), 5 °C/min to 230 °C (5 min), and 10 °C/min to 280 °C (5 min). The electron-impact mass spectra were generated at 70 eV, with a scan range from 30 to 600 *m/z*, the ion source temperature was 230 °C, and the MS interface temperature was 250 °C.

### 3.8. Statistical Analysis

Statistical significance was carried out by applying one-way ANOVA followed by Duncan’s test with the acceptance level of significance *p* = 0.05, using SPSS version 22.0 (IBM Corp., Armonk, NY, USA). Logistic analysis was performed using Origin version 2017 software (OriginLab., Northampton, MA, USA) and GraphPad Prism 7 software (GraphPad Software, Inc., La Jolla, CA, USA).

## 4. Conclusions

To the best of the authors’ knowledge, this is first report that investigates the volatile chemical composition of the SFE-CO_2_ extract from *B. frutescens* leaves. β-Caryophyllene, α-caryophyllene, δ-cadinene, eucalyptol and terpineol are the major compounds of the extract. The extract presented antifungal activity against *P. camelliae* and *C. gloeosporioides*. Terpineol, linalool, terpinen-4-ol and eucalyptol are the main active ingredients. Therefore, the SFE-CO_2_ extract from *B. frutescens* leaves could be a potential alternative to traditional synthetic chemical fungicides of tea plant pathogens (*P. camelliae* and *C. gloeosporioides*).

## Figures and Tables

**Table 1:** Chemical compounds identified in the volatile supercritical fluid extraction (SFE-CO_2_) extract of B. *frutescens*.

Peak NO	Compound Name	RI _(*C*)_^1^	RI _(*L*)_^2^	Relative Area (%) ^3^	Identification Methods
1	α-Pinene	940	944	2.11	RI, MS, STD ^4^
2	β-Pinene	987	979	5.21	RI, MS, STD
3	o- Cymene	1044	1042	1.18	RI, MS
4	Eucalyptol	1049	1047	5.46	RI, MS, STD
5	γ-Terpinene	1074	1063	2.49	RI, MS, STD
6	α-Terpinolene	1104	1097	0.38	RI, MS
7	Linalool	1115	1104	1.47	RI, MS, STD
8	Terpinen-4-ol	1194	1182	0.83	RI, MS, STD
9	Terpineol	1209	1200	4.54	RI, MS, STD
10	Epicubebol	1433	1494	0.19	RI, MS
11	β-Caryophyllene	1448	1439	28.05	RI, MS, STD
12	α-Caryophyllene	1485	1452	24.02	RI, MS, STD
13	α-Muurolene	1520	1500	1.60	RI, MS
14	δ-Guaiene	1523	1502	0.57	RI, MS
15	δ-Cadinene	1536	1524	6.29	RI, MS
16	trans-Calamenene	1542	1527	1.28	RI, MS
17	6-epi-Shyobunol	1548	1548	0.29	RI, MS
18	Cadine-1,4-diene	1552	1546	0.64	RI, MS
19	Isoshyobunone	1605	1562	1.63	RI, MS
20	Caryophyllene oxide	1610	1581	0.70	RI, MS
21	Unkown	1618	-	0.77	RI, MS
22	Humulene epoxide II	1624	1616	0.86	RI, MS
23	Unkown	1633	-	0.38	RI, MS
24	α-Acorenol	1643	1630	0.73	RI, MS
25	Cubenol	1648	1637	2.81	RI, MS
26	Longifolenaldehyde	1656	1631	2.40	RI, MS
27	β-Acorenol	1661	1649	2.11	RI, MS
Total				98.99	

^1^ RI **_(*C*)_**: Retention index was calculated with relative to the homologous series of (C5–C36) alkanes under the same operating conditions. ^2^ RI **_(_*_L_*_)_**: Retention index reported in the relative literature for equivalent capillary column. ^3^ Relative Area (%): Relative area (peak area relative to the total peak area); ^4^ STD: co-injection with standard compound have the same mass spectrum (MS) and RI.

**Table 2:** Contact antifungal toxicity of *B. frutescens* leaf SFE-CO_2_ extracts against *P. camelliae* and *C. gloeosporioides*.

Content (μg/mL)	Inhibitory Rate (%) ^1^	
	*P. camelliae*	*C. gloeosporioides*
50	66.63 ± 2.05 ^a,2^	64.59 ± 1.59 ^a^
25	65.77 ± 1.35 ^a^	63.94 ± 0.39 ^a^
12.5	63.09 ± 2.84 ^a^	61.69 ± 0.46 ^a^
6	54.11 ± 1.18 ^b^	53.05 ± 0.87 ^b^
3	32.48 ± 4.37 ^c^	40.29 ± 1.65 ^c^
Control	0.00 ± 1.08 ^d^	0.00 ± 0.16 ^d^
MIC_50_	5.11 μg/mL	4.79 μg/mL

^1^ Mean (±standard error) of three replicates for each sample. ^2^ Percentage values followed by the same letter are not significantly different in the same group at *p* ≤ 0.05 (Duncan’s test).

**Table 3:** Contact antifungal toxicity of the four major chemical compounds of *B. frutescens* leaf SFE-CO_2_ extracts against *P. camelliae* and *C. gloeosporioides*.

Compound	*P. camelliae*				*C. gloeosporioides*			
	MIC_50_ (μL/mL)	95% CI ^1^ (μL/mL)	Chi Square (χ^2^)	R^2^	MIC_50_ (μL/mL)	95% CI (μL/mL)	Chi Square (χ^2^)	R^2^
Terpineol	0.69	0.66–0.72	1.40	0.999	0.62	0.49–0.75	9.88	0.988
Linalool	0.73	0.63–0.82	10.61	0.994	0.93	0.74–1.13	12.32	0.990
Terpinen-4-ol	0.86	0.69–1.09	25.82	0.986	0.94	0.66–1.21	23.90	0.987
Eucalyptol	2.79	2.62–2.97	6.50	0.980	2.18	2.05–2.3	5.07	0.981
Carbendazim	9.70	9.41–10.01	0.55	0.999	0.095	0.091–0.100	1.22	0.999

^1^ 95%CI: 95% confidence interval for each of three replicates for each median inhibitory concentration (MIC_50_) value.

## Supplementary Materials

The following are available online at https://www.mdpi.com/2223-7747/9/9/1119/s1, Figure S1: The total ion chromatogram of volatile fraction of *Baeckea frutescens* L (SFE-CO2) extract by GC-MS.
