# Supplementary material for: Chemical Composition of a Supercritical Fluid (Sfe-CO2) Extract from Baeckea frutescens L. Leaves and Its Bioactivity Against Two Pathogenic Fungi Isolated from the Tea Plant (Camellia sinensis (L.) O. Kuntze)
Source: Plants (Basel). 2020 Aug 29;9(9):1119. doi: 10.3390/plants9091119 (PMC7569807; doi:10.3390/plants9091119)
Supplement: Supplementary file 1 [file plants-09-01119-s001.pdf]

plants

MDPI

Supplementary Materials

Chemical composition of a supercritical fluid (SFE-CO2) extract from *Baeckea frutescens* L. leaves and its bioactivity against two pathogenic fungi isolated from the tea plant [*Camellia sinensis* (L.) O. Kuntze]

Hao Jiang [1,†], Mengting Zhang[1,†], Li Qin[2], Dongxu Wang[3], Feng Yu[1], Wenhui Liang[4], Daniel Granato [5,*] and Chuankui Song [1,*]

[1] State Key Laboratory of Tea Plant Biology and Utilization, Anhui Agricultural University, 130 West Changjiang Road, Hefei 230036, China; ahjh88@163.com (H.J.); mrs123zhang@163.com (M.Z.); zixinyu147258@163.com (F.Y.)
[2] Anhui Provincial Key Laboratory of Microbial Pest Control, Anhui Agricultural University, 130 West Changjiang Road, Hefei 230036, China; qinli@ahau.edu.cn
[3] School of Grain Science and Technology, Jiangsu University of Science and Technology, Zhenjiang 212003, China; justwdx@163.com
[4] Guangxi Forestry Research Institute, Guangxi Engineering and Technology Research Center for Woody Spices, Guangxi Key Laboratory for Cultivation and Utilization of Special Non-Timber Forest Crops, 23 Yongwu Road, Nanning 530002, China; 723746615@qq.com
[5] Food Processing and Quality, Natural Resources Institute Finland, Tietotie 2, 02150, Espoo, Finland
* Correspondence: Daniel.granato@luke.fi (D.G.); sckfriend@163.com (C.S.)
† These authors contributed equally to this work.
* Correspondence: Daniel.granato@luke.fi (D.G.); sckfriend@163.com (C.S.)

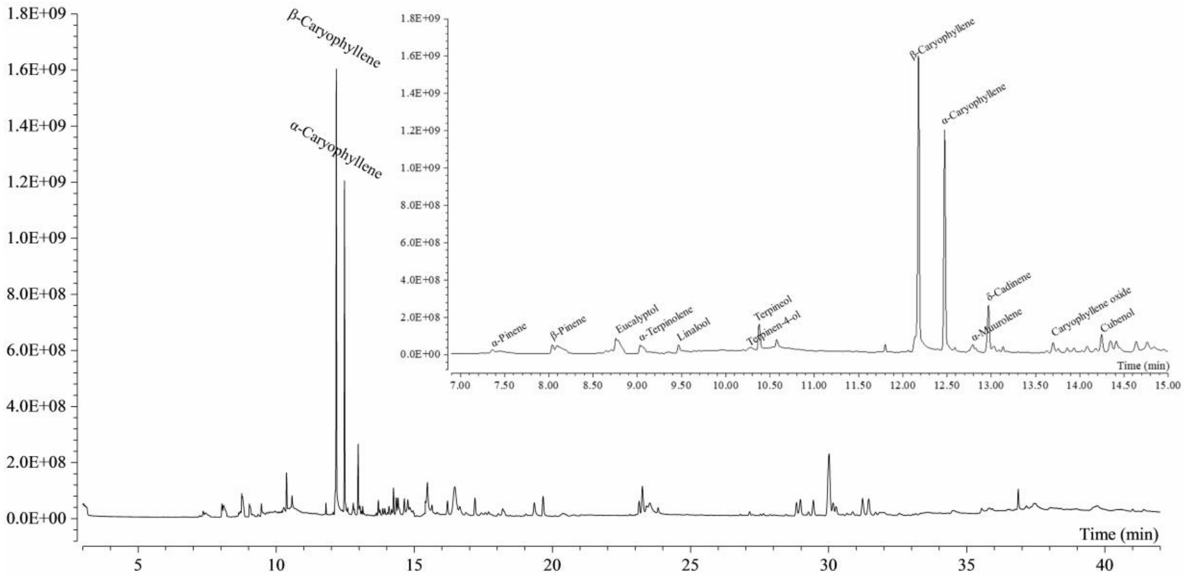

Figure S1. The total ion chromatogram of volatile fraction of *Baeckea frutescens* L (SFE-CO2) extract by GC-MS.

© 2020 by the authors. Submitted for possible open access publication under the terms and conditions of the Creative Commons Attribution (CC BY) license (http://creativecommons.org/licenses/by/4.0/).
